# Use of Atezolizumab for Bladder and Non–Small Cell Lung Cancers

**Published:** 2017-07-01

**Authors:** Kayla Faith Moore

**Affiliations:** St. Francis Cancer Center, Greenville, South Carolina

Atezolizumab (Tecentriq) is an immunotherapy agent with activity across a broad range of tumor types including bladder and non–small cell lung cancer (NSCLC). Recently, immunotherapy has shown a promising avenue of clinical research in both of these disease states.

## BACKGROUND ON BLADDER AND LUNG CANCERS

Bladder cancer is the sixth most common cancer in the United States, with an estimated 76,960 new cases diagnosed in 2016 (American Cancer Society, 2016). Of these patients, 16,390 deaths were estimated to have occurred, with men being more likely to be affected than women. The 5-year relative survival rate for all stages combined is 77% (Howlader et al., 2016). However, survival rates depend on many factors, including the histology and stage of bladder cancer diagnosed. For patients with bladder cancer that is invasive but not yet spread outside the bladder, the 5-year survival rate is 70%. For patients with bladder cancer that extends through the bladder to the surrounding tissue and/or organs, the 5-year survival rate is 34%.

A cisplatin-based chemotherapy regimen followed by surgical removal of the bladder or radiation therapy and concomitant chemotherapy is currently the standard treatment for patients with invasive bladder cancer. The recent advances in immunotherapy have expanded treatment options for patients with metastatic disease, not at the time of cystectomy.

Lung cancer is the second most common diagnosed cancer in both men and women and still remains the leading cause of cancer-related death globally (Rittmeyer et al., 2017). The 5-year survival rate for lung cancer is 17%, with only 16% being diagnosed at a localized stage.

Docetaxel has been the standard of care for disease that has relapsed or is refractory to platinum therapy; however, its efficacy is sometimes offset by the toxicities associated with its use. Recent studies have now shown that immunotherapy can induce a durable response and improve overall survival for this disease (Rosenberg et al., 2016).

## PHARMACOLOGY

Atezolizumab is a humanized monoclonal antibody that acts directly against the programmed cell death ligand 1 (PD-L1). Atezolizumab binds to PD-L1 and blocks its binding and activation to programmed cell death protein 1 (PD-1) and B7-1 expressed on activated T cells. By inhibiting PD-L1, atezolizumab enables the activation of T cells, restoring their ability to effectively detect and attack tumor cells. 

## CLINICAL STUDIES

Atezolizumab was first evaluated in metastatic urothelial bladder cancer in a phase I study of 67 patients, 62 of whom had previous platinum-based chemotherapy and 48 of whom had had at least 2 prior systemic therapy regimens (Powles et al., 2014). Objective response rates were 43% for patients who exhibited high or moderate PD-L1 group expression and 11% for those with low or absent levels of PD-L1. Complete responses were noted in 7% of patients who had immunohistochemistry (IHC) 2/3 tumors and a minimum of 12 weeks of follow-up. The results of this study allowed atezolizumab to receive Breakthrough Designation status by the US Food and Drug Administration (FDA) in June 2014.

Conducted in 2015, an expansion of this study evaluated 87 patients (Petrylak el al., 2015). Objective response rates were 50% for the high and moderate PD-L1 group and 17% in the low and absent PD-L1 group. Median progression-free survival was 6 months in the high/moderate PD-L1 group and 1 month in the low/absent PD-L1 group. Overall survival was not reached in the high/moderate group and was approximately 7 months in the low/absent group.

A phase II study assessed the treatment with atezolizumab in patients with metastatic urothelial carcinoma who failed to respond to platinum-based chemotherapy (Rosenberg et al., 2016). A total of 19% of patients had disease progression following prior platinum-containing neoadjuvant or adjuvant chemotherapy, with 41% of patients receiving at least 2 prior systemic regimens in the metastatic setting. Patients received 1,200 mgof atezolizumab by intravenous infusion every 3 weeks until unmanageable toxicity or clinical disease progression. Outcome measures included confirmed objective response and duration of response.

Compared with a historical control, overall response rates of 10%, atezolizumab significantly improved objective response rates, with a confirmed objective response of 14.8% for all patients. A total of 84% of responders continued to respond after 1 year of follow-up. Patients with tumors categorized as IC 2/3 (PD-L1 expression ≥ 5%) had a median overall survival of 11.4 months vs. IC1/IC0 of 6.7 and 6.5 months, respectively. The 12-month landmark overall survival rate averaged around 41% across prespecified immune cell groups. The FDA approved atezolizumab based on these results for the treatment of patients with bladder cancer with locally advanced or metastatic urothelial carcinoma whose disease progressed during/after platinum-based chemotherapy or within 12 months of neoadjuvant treatment with platinum-based chemotherapy.

For the treatment of NSCLC, a phase III trial exhibited that atezolizumab treatment resulted in significant improvement in overall survival compared with docetaxel in previously treated patients with advanced-stage NSCLC who had disease progression during or after platinum-based chemotherapy (Rittmeyer et al., 2017). Patients received atezolizumab at 1,200 mg or docetaxel at 75 mg/m² every 3 weeks. Endpoints included overall survival in the intention-to-treat (ITT) population and PD-L1 expression population TC1/2/3 or IC1/2/3.

Overall survival was significantly longer with atezolizumab vs. docetaxel in the intention-to-treat and PD-L1 expression populations. Median overall survival with atezolizumab was 13.8 months vs. 9.6 months with docetaxel in the ITT population. Overall survival in the TC1/2/3 or IC1/2/3 with atezolizumab was 15.7 months vs. 10.3 months with docetaxel. Median overall survival was also improved with atezolizumab (12.6 months) vs. docetaxel (8.9 months) in the subgroup of patients with PD-L1 low or undetectable. Also, fewer patients had treatment-related adverse events in the atezolizumab group (15%) vs. docetaxel group (43%). This trial showed a clinically meaningful survival benefit with atezolizumab over docetaxel in previously treated patients with NSCLC.

## ADVERSE EFFECTS

In general, atezolizumab has been well tolerated across clinical trials. Most adverse events have been mild to moderate. A phase II trial evaluated 310 patients with inoperable locally advanced or metastatic urothelial carcinoma whose disease had progressed after platinum-based chemotherapy for safety and adverse events. The percentage of patients reporting adverse events of any grade was 97%, with 55% having grade 3 or 4 adverse events (Rosenberg et al., 2016). The most common adverse events included fatigue (49%), nausea (26%), decreased appetite (27%), pyrexia (22%), and diarrhea (20%). High-grade adverse events (grade ≥ 3) included anemia (9%), fatigue (6%), dyspnea (4%), nausea (2%), and hypertension (2%). A total of 69% of patients experienced a treatment-related adverse event, with 16% experiencing a high-grade treatment-related adverse event. The most common treatment-related adverse events included fatigue (30%), nausea (14%), decreased appetite (12%), pruritus (10%), pyrexia (9%), diarrhea (8%), rash (7%), arthralgia (7%), and vomiting (6%).

Not only did Rittmeyer and colleagues (2017) find that atezolizumab resulted in clinically relevant improvement of overall survival vs. docetaxel in previously treated NSCLC, regardless of PD-L1 expression or histology, but that atezolizumab had a favorable safely profile. There were fewer treatment-related adverse events with atezolizumab than with docetaxel, including grade 3 or 4 events (15% of 609 patients vs. 43% of 578 patients). Of the 609 patients in the safety analysis, immune-mediated adverse events reported with atezolizumab included pneumonitis (1% at any grade; < 1% grade 3), hepatitis (< 1%), and colitis (< 1%). Adverse events leading to discontinuation of treatment occurred in 8% of atezolizumab patients and 19% of docetaxel patients. There were no deaths related to atezolizumab; however, there was one death related to docetaxel.

## THERAPEUTIC ROLE

Evidence has shown that PD-1 and PD-L1 receptor pathways are valid targets in the treatment of many malignancies, including metastatic bladder cancer and NSCLC. Although atezolizumab has shown promising results in locally advanced and metastatic bladder cancer, there has been no definite proof of a survival benefit with atezolizumab treatment after a patient has experienced progression during or after platinum-based chemotherapy. Atezolizumab is also being examined in localized bladder cancer; however, currently cisplatin-based chemotherapy is the only systemic therapy proved to extend survival in this patient population.

Targeting PD-1 and PD-L1 receptor pathways with immunotherapy agents such as atezolizumab has also shown promising results and clinically relevant improvements in overall survival compared with docetaxel in patients with NSCLC. A phase I study (Horn et al., 2015) showed durable antitumor responses in NSCLC as well as an association of PD-L1 expression of tumor cells and tumor-infiltrating immune cells with patients who had an objective response. In the phase II POPLAR study, atezolizumab improved overall survival in patients NSCLC who had been previously treated with docetaxel. The phase III OAK trial that evaluated safety and efficacy of atezolizumab in patients with NSCLC found a clinically meaningful survival benefit over docetaxel in previously treated patients regardless of PD-L1 expression or histology. These data suggest that atezolizumab is a new treatment option for patients with advanced NSCLC whose disease has progressed during or after platinum-based chemotherapy. 

## IMPLICATIONS FOR THE ADVANCED PRACTITIONER

The new development of antibodies that target PD-L1 represents an important advancement in the management of metastatic bladder cancer; however, there is no definite proof-of-survival benefit with atezolizumab treatment in the second line for this patient population. Until further investigation, atezolizumab is an option for the treatment of locally advanced or metastatic urothelial carcinoma, including bladder cancer and other urinary cancers, in patients whose disease progresses during or after platinum-containing chemotherapy.

Clinical studies have shown that atezolizumab has a proven overall survival benefit in NSCLC as well as a manageable safety profile. In October 2016, the FDA approved atezolizumab for the treatment of patients with metastatic NSCLC whose disease progressed during or after platinum-containing therapy. Atezolizumab joins other immunotherapy agents, which are becoming an important component for patients with NSCLC when other first-line treatment options do not work.

The side-effect profile for this drug remains favorable, making it beneficial in certain patient populations who may not be able to tolerate other treatment options. Immunotherapy agents, however, are still associated with potential adverse reactions and side effects. During treatment with these agents, patients’ liver function (aspartate transaminase, alanine transaminase, and bilirubin) should be monitored at baseline and periodically, as immune hepatitis can occur. Immune-mediated pancreatitis associated with atezolizumab occurred in less than 1% of patients in clinical trials; however, an interruption in therapy may be warranted should this occur. Thyroid function should also be assessed at baseline and periodically in patients starting atezolizumab. In general, diarrhea and colitis were reported in 18% to 19% of patients across clinical trials, and immune-mediated colitis or diarrhea with no clear alternate etiology occurred in a total of 8 patients. Caution should be used with atezolizumab in patients with inflammatory bowel disease such as ulcerative colitis or Crohn’s disease. Corticosteroids should be administered to patients who experience grade 2 or higher diarrhea or colitis.

Finally, it will also be important to discuss cost with patients before starting treatment with atezolizumab, as this drug remains very expensive. See the Table above for dosage and administration guidelines for atezolizumab.

**Table:**
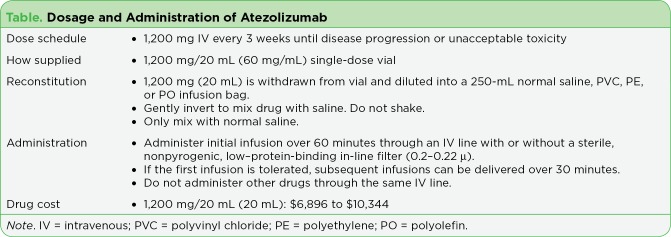
Dosage and Administration of Atezolizumab
